# Endoscope-assisted scleral buckle procedure

**DOI:** 10.1186/s40942-020-00260-x

**Published:** 2020-11-11

**Authors:** Sean M. Platt, Andrew J. Barkmeier

**Affiliations:** 1 Department of Ophthalmology, Mayo Clinic, 200 First St. SW, Rochester, MN 55905 USA; 2 Retina Associates of Cleveland, Inc., Cleveland, OH USA

**Keywords:** Endoscope, Scleral buckle, Retinal detachment, Peters' anomaly

## Abstract

**Background:**

Retinal reattachment surgery requires clear visualization of the posterior segment for optimal outcomes. Select patients may benefit most from primary scleral buckling without vitrectomy, but lack adequate posterior segment ophthalmoscopic visualization to use standard techniques.

**Case presentation:**

The authors describe a retinal reattachment technique utilizing endoscope-assisted visualization to perform a primary scleral buckle procedure for a 34yo female with Peters’ Anomaly and a macula-sparing retinal detachment. Retinal reattachment was achieved with a single procedure and she remained stable with preservation of baseline visual acuity at 30 months follow-up.

**Conclusion:**

In cases where a primary scleral buckle procedure is the preferred retinal detachment repair technique but posterior segment visualization is limited, intraoperative fundus examination, cryotherapy administration, and scleral buckle positioning can be facilitated with intraocular endoscopy.

## Background

Retinal reattachment surgery requires clear visualization of the posterior segment for optimal outcomes. At times, opacification of the anterior segment ocular media must be addressed during vitreoretinal surgery, either via techniques aiming to clear the media (e.g. lensectomy, corneal epithelial scraping, temporary keratoprosthesis), or potentially through the use of endoscopic visualization to bypass the anterior segment.

We describe a technique that was developed to perform retinal reattachment surgery on a 34 year old female with Peters’ Anomaly, microcornea, nystagmus, and ocular hypertension. She presented with acute vision changes and was diagnosed with macula-sparing retinal detachment on B-scan ultrasonography. Visual acuity was preserved at her 20/300 baseline. She was monocular following multiple surgeries in her fellow eye including trabeculectomy, penetrating keratoplasty, multiple retinal detachment surgeries, and eventual enucleation. A primary scleral buckle operation was preferred for several reasons, but anterior segment pathology including corneal scarring, cataract, and iris anomalies did not permit sufficient ophthalmoscopic visualization of the peripheral retina.

## Surgical technique

Prior to surgery, meticulous ultrasonography was performed with attention to the boundaries of detached retina, as well as to any areas concerning for vitreoretinal pathology. This step is critical for determining the optimal trocar cannula placement to facilitate localization and treatment of retinal breaks, and to minimize the risk of crystalline lens trauma from the endoscope. A standard 360° conjunctival peritomy was fashioned and a single 23-gauge trocar cannula was placed based upon the ultrasound findings. A 23-gauge endoscope (Endo Optiks, BVI Medical, Waltham, MA, USA) was introduced and the peripheral retina is inspected endoscopically with primarily in-and-out movements of the endoscope. Sweeping motions of an extended endoscope would risk creation of iatrogenic retinal breaks in a vitreous-filled eye. Retinal breaks were then marked externally with a marking scleral depressor for future reference during scleral buckle placement. The cryotherapy probe was then placed over scleral marks and all retinal breaks are treated under direct visualization (Additional file 1: Video S1).

Although various scleral buckle elements and placement techniques may be used in an endoscope-assisted scleral buckling procedure, we employed the following technique to ensure appropriate scleral buckle height in this context of limited posterior segment visualization. We initially placed a 275-style solid silicone element around the eye beneath all four rectus muscles, trimming the ends to leave them gently apposed. Next, a 5 mm segment of one end was measured and removed. A 5-0 mersilene suture was placed in each quadrant with 7.5 mm anteroposterior spacing and attention to supporting the posterior edge of previously-marked retinal breaks. The buckle ends were then re-apposed and affixed with two 5-0 mersilene sutures. This technique offers consistent and predictable imbrication without the need for serial adjustments. The subconjunctival space was irrigated with antibiotic solution and the conjunctiva was sutured closed.

## Discussion

In this case, an otherwise uncomplicated retinal detachment was successfully treated while avoiding premature cataract extraction and post-vitrectomy cataractogenesis, destabilization of ocular hypertension already requiring maximum medical therapy, or extensive anterior segment surgery (keratoprosthesis would be problematic due to microcornea). Procedure-specific risks include both iatrogenic crystalline lens trauma and endoscope-related retinal breaks in a vitreous-filled eye. The magnitude of these risks would depend upon both the clinical scenario and the surgeon’s comfort with endoscopy. In the context of significantly limited surgical visualization, it is important to note that the consequences of any intra-operative complication would potentially be magnified.

Endoscopy plays an important niche role in the management of vitreoretinal pathologies. It has been successfully employed to facilitate vitrectomy procedures when posterior segment visualization is limited due to anterior segment media opacity [1], when retinal breaks remain undetected with standard techniques [2], in trauma [3], endophthalmitis [4], pediatric vitreoretinal surgery [5], as well as for specific techniques such as suturing of intraocular lenses [6].

The technique described herein for endoscope assisted scleral buckle procedures is relatively straightforward for vitreoretinal surgeons with modest endoscope experience. Simultaneous bimanual control of both the endoscope and scleral depression/cryotherapy are relatively intuitive for an experienced vitreoretinal surgeon. Although there are a limited number of clinical scenarios for which this procedure would be most appropriate, we propose that it is an important option for vitreoretinal surgeons to consider as a part of their surgical armamentarium for carefully selected patients.

## Supplementary information

**Additional file 1: Video S1.** Endoscope-assisted scleral buckle surgical video.

## Data Availability

Data sharing is not applicable to this article as no datasets were generated or analyzed during the current study.
